# Dynamic Facial Reanimation in an Overweight Patient and with Significant Comorbidities: An Objective Analysis of Labbè Technique

**DOI:** 10.1055/s-0041-1736668

**Published:** 2021-12-15

**Authors:** Ricardo Horta, Francisca Frias, Diogo Barreiro, Ana Gerós, Paulo Aguiar

**Affiliations:** 1 Department of Plastic, Reconstructive and Maxillo-Facial Surgery, and Burn Unity, Centro Hospitalar de São João, Porto Medical School, Porto, Portugal; 2 FEUP - Faculdade de Engenharia da Universidade do Porto, INEB - Instituto Nacional de Engenharia Biomédica, i3S - Instituto de Investigação e Inovação em Saúde, Porto, Portugal; 3 FMUP - Faculdade de Medicina da Universidade do Porto, INEB - Instituto Nacional de Engenharia Biomédica, i3S - Instituto de Investigação e Inovação em Saúde, Porto, Portugal

**Keywords:** facial palsy, facial reanimation, labbè technique, overweight patient

## Abstract

Gracilis free muscle transfer is considered the gold standard technique for facial reanimation in cases of facial palsy. However, it is limited by its long operative and recovery times, the need for a second surgical site, and its outcomes that can sometimes show midfacial bulk and oral commissure malposition. Facial reanimation with lengthening temporalis myoplasty (LTM)—Labbé technique— carries the advantage of having a shorter surgical time, a faster recovery, and being a less invasive surgery. Almost all patients included in studies of LTM were evaluated by subjective methods, and very little quantifiable data was available. A 64-year-old woman presented with long-standing incomplete right facial palsy secondary to acoustic neuroma surgery. Since she was overweight (body mass index [BMI]: 43.9) and had several cardiovascular comorbidities (hypertension, dyslipidemia), she was not a good candidate for gracilis free muscle transfer. She was submitted to facial reanimation with LTM. Fourteen months after surgery, she presented excellent facial symmetry, both at rest and in contraction, while smiling. She was evaluated with the Facegram-3D, a technology that we have developed for dynamic evaluation of facial muscle contraction. The analysis showed symmetry at rest and contraction, according to Terzis and Noah. Regarding vertical and horizontal displacement, the postoperative movement was synchronized and with less fluctuations when compared with the preoperative period. Notably, the anatomical pair's trajectories were smoother. Similar velocity profiles were found between anatomical pairs, with less abrupt changes in velocity values, further supporting improved movement control. Comparing the symmetry index, which takes a theoretical maximum of 1.0 for perfect 3D symmetry, its value was 0.56 for the commissures and 0.5 for the midpoints in the preoperative period, having improved to 0.91 and 0.82, respectively, 3 months postoperatively. Good aesthetic and functional results were achieved using the Labbè technique. LTM is a good option in cases of long-standing facial paralysis, if the patient desires a single-stage procedure with almost immediate dynamic function. Moreover, this technique assumes extreme importance in facial reanimation of patients of advanced age, overweight, or those who have several comorbidities.

Psychological distress is well-documented in people with facial disfigurement and facial palsy and can have a tremendous impact on the affected individuals, causing psychological and physical fatigue. 1 To overcome this psychological distress, surgical procedures can be performed. Free flap muscle transfer is the preferred technique in long-standing facial palsy for restoring symmetry both at rest and when smiling. 2 Gracilis free muscle transfer is considered one of the best options but its use can sometimes result in suboptimal aesthetical outcomes due to midfacial bulk or oral commissure malposition. 3 Moreover, it often involves a multistage procedure in which cross-face grafts are used in advance of the free flap (except if direct neurotization is performed, e.g., with the masseteric branch), involving long operative and recovery times as well as a second surgical site. 4 For some patients, the delay and the need for multiple extensive procedures and long-term physiotherapy make this option not possible. 5 Other procedures should then be considered such as Labbé technique. This procedure consists of a one-time surgery in which an orthodromic temporalis tendon transfer can be performed in a minimally invasive manner, eliminating the facial asymmetry. 5

## Clinical Report



**Video 1** Preoperative Facegram video analysis.


**Video 2** Postoperative Facegram video analysis.


**Fig. 1:**
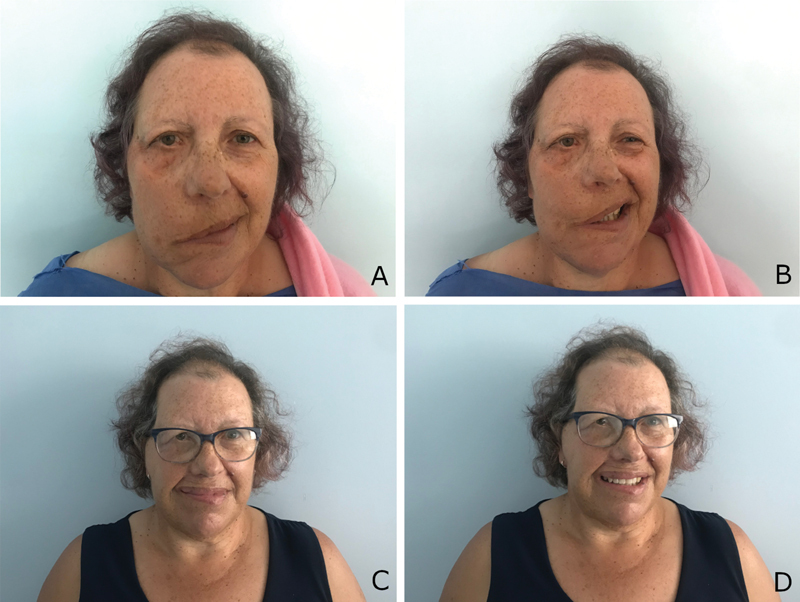
A 63-year-old woman with right facial palsy secondary to the removal of an acoustic neuroma was submitted to a facial reanimation with lengthening temporalis myoplasty (LTM)—Labbé technique. Preoperative views: ( **A** )—frontal view at rest; ( **B** )—frontal view, smiling. Postoperative views: ( **C** )—frontal view at rest; ( **D** )—frontal view, smiling.

A 64-year-old woman presented with long-standing, incompletely recovered right facial palsy secondary to acoustic neuroma surgery, with weak commissure excursion on the right side. Since she was overweight (body mass index [BMI]: 43.9) and had several cardiovascular comorbidities (hypertension, dyslipidemia), she was not the perfect candidate for the gold standard technique—gracilis free muscle transfer. She was submitted to facial reanimation with lengthening temporalis myoplasty (LTM)—Labbé technique ( Fig. 1A and B ).

An incision extending from the preauricular crease into the temporal hair was performed to access the temporalis muscle. Dissection was performed deep to the periosteum of the zygomatic arch, and the temporalis muscle was trailed from the inferior surface of the arch to its insertion into the coronoid process. The coronoid was transected along the attached temporalis tendon, preserving as much of the tendon as possible. The flap was then tunneled to a separate incision, made at the melolabial crease, and sutured to the lip musculature and some deep dermis. The location of this attachment is intended to produce maximum symmetry as determined before surgery. A titanium plate was afterward placed in the upper right eyelid to correct the lagophthalmos. Botulinum toxin was also applied to correct residual asymmetry of the lower lip (contralateral lip depressors).

**Fig. 2:**
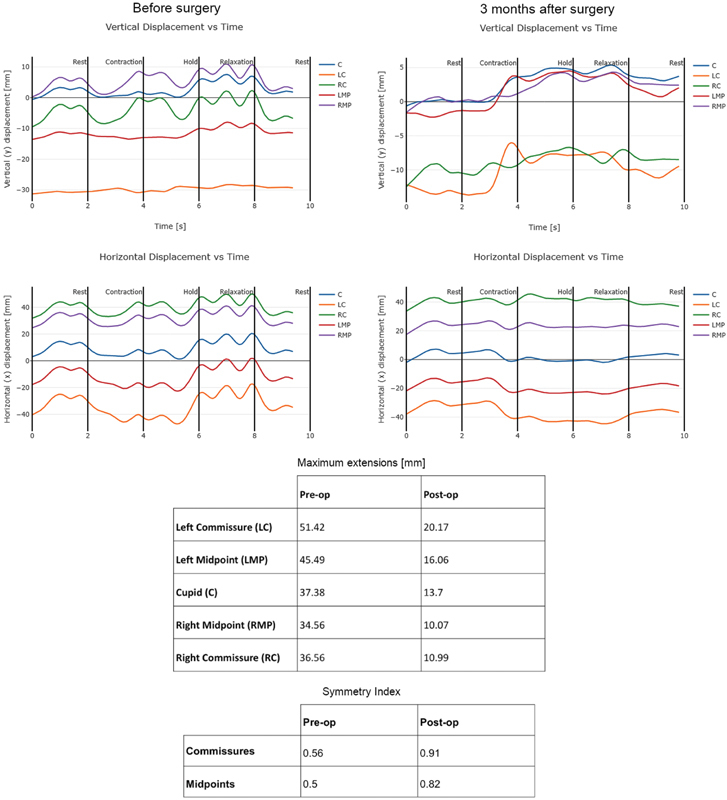
Vertical and horizontal displacement versus time pre- and postop. Maximum extensions and symmetry index pre- and postop.

Fourteen months after surgery, she presented excellent facial symmetry, both at rest and in contraction, while smiling ( Fig. 1C and D ) with good formation of a nasolabial fold. She was evaluated with the Facegram system, a technology developed by us for dynamic evaluation of facial muscle contraction. 6 7 This system allows 3D spatiotemporal analysis, compiled in a set of distilled measurements with clinical usefulness for a complete characterization of facial movements (the Facegram report). The postoperative analysis showed symmetry at rest and contraction, according to Terzis and Noah. Regarding vertical and horizontal displacement, the postoperative movement was synchronized and with less fluctuations when compared with the preoperative period (see Supplemental Videos 1 and 2 [online only], which demonstrates respectively preoperative and postoperative Facegram analysis). Notably, the anatomical pair's trajectories were smoother and more consistent over time, suggesting a significant improvement in controlling facial ( Fig. 2 ). Additionally, in postoperative movement, similar velocity profiles were found between anatomical pairs, with less abrupt changes in velocity values, further supporting improved movement control. The extent of movement of both commissures and midpoints on the nonparalyzed side was also inferior after surgery, meaning more balance and less need of compensatory movement.

Comparing the symmetry index, which takes a theoretical maximum of 1.0 for perfect 3D symmetry, its value was 0.56 for the commissures and 0.5 for the midpoints in the preoperative period, having improved to 0.91 and 0.82, respectively, 3 months postoperatively.

## Discussion

Regional muscle transposition and free muscle transfer are the two principal methods of dynamic muscle transfer for facial reanimation. 8 Gracilis free muscle transfer is the preferred technique, because of its consistent anatomy, its good size for facial functional restoration, single innervated motor nerve and the potential of adjusting the vector of smile and commissure position. 4 Operation time for free functional muscle transfer for facial reanimation in high-volume centers is around 3 to 4 hours with maximal success rates when trigeminal nerve branches are used for innervation, even in older patients. Neurotization time for these free muscle transfers averages between 2 to 4 months, while a 12- to 24-month period is required when a cross-face graft is used. 9

On the other hand, regional muscle procedures are limited by anatomic constraints of size and vectors, the results being not consistent. However, they are technically less demanding, provide immediate reanimation, and can be appropriate in debilitated patients or patients who cannot wait for a period of free muscle transfer neurotization. They also avoid distant donor site morbidity.

There are currently very few randomized controlled trials (RCTs) and controlled clinical trials (CCTs) regarding facial reanimation surgery, and only very weak evidence is available to support the use of one type of surgery over another. A systematic review to compare the outcomes of reconstructive surgery for long-standing facial paralysis by gracilis free flap transfer versus LTM was performed by Bos et al 10 Gracilis flap transfer involving masseteric nerve reinnervation achieved greater commissural displacement than a cross-facial nerve graft reinnervation. Patients with double innervation had similar results to those who had surgery involving only masseteric nerve reinnervation. Patients operated by the LTM achieved less lateral movement of the commissure, with controversial evidence of spontaneity (only “automatic”). This review also suggested that LTM achieves results that are at least equal to those obtained with gracilis transfer, but LTM is a less extensive procedure that provides excellent aesthetic results, quicker recovery, earlier visible result, low donor site morbidity, without the need for more than one operation. It also results in very high rates of spontaneous smiling and does not require microvascular anastomosis; therefore, it should be considered a suitable alternative to gracilis free muscle transfer. 11 However, it remains considerably less common than free muscle flaps.

Facial reanimation surgery has a long learning curve, and experience is necessary to achieve good results. Surgical teams all over the world have reported significant improvement in outcomes over time, using different techniques.

A significant disadvantage of the Gillies technique and its modifications is that temporal hollowing occurs as a result of muscle harvesting, thus exaggerating facial asymmetry. LTM according to Labbé avoids temporal hollowing by two maneuvers: preserving the superficial temporal fat pad, and dissecting just above the deep temporal fascia. The muscle should be released from the temporal fossa with care for the neurovascular pedicle. 10 In our case, some minor temporal hollowing was seen but can still be improved in the future.

The tendon, after being stripped from the coronoid process, is advanced to the nasolabial incision, being sutured at the commissure to create symmetry at rest, and the anterior and longest part of the tendon can be also attached at the alar base to correct the nasal scoliosis. In our patient, some upper lip eversion was seen, suggesting a more superficial insertion of the temporalis muscle.

Because of technical issues such as ensuring that the fascia of the temporalis muscle tendon reaches the nasolabial fold, performing an osteotomy of the coronoid process and zygomatic arch, which may seem complicated, and facial incisions, many surgeons do not consider LTM an option, except for older people who are not good candidates for free muscle transfer or for people who desire less extensive surgery. 12 However, the procedure is fairly simple for a skilled surgeon with good knowledge of the local anatomy. 13

Almost all patients included in studies of LTM were evaluated by subjective methods, and very little quantifiable data was available. Our patient was evaluated using a standardized measuring scale after standardized physical therapy postoperatively and more than 1 year of follow-up. However, it should be noted that objective measurements do not necessary provide meaningful information about the quality of a reanimated smile. Indeed, subjective evaluation can sometimes provide more information about outcomes than simply objective data.

In this case, since the patient had already several medical conditions and obesity (with a higher risk of seroma and donor-site complications), a free muscle transfer was not an option due to a higher risk of medical and surgical complications. The regional muscle selected for transposition was the temporalis muscle and Labbè technique was used. Like in all dynamic muscle transfers, the temporal muscle modifies its function because it is completely mobilized toward another effector: the labial commissure, but due to cerebral plasticity, it loses its masticatory function and acquires its new smile function. 14 Almost all the patients submitted to Labbè technique obtain an “automatic” spontaneous smile, when compared with the ones submitted to the procedure using gracilis free muscle flaps, in which approximately only two-thirds of patients achieved a spontaneous smile. 10 This finding was unexpected, because the mechanism of achieving a spontaneous smile, namely, cerebral plasticity, is the same in both procedures. The use of different kinds of postoperative speech therapy may have contributed to these findings. The limitations of LTM are the risk of overcorrection and consequent creation of facial asymmetry, the unsightly defect created in the temporal region by removing the muscle from its fan-shaped origin on the squamous portion of the temporal bone. Compared with the traditional retrograde temporal muscle procedure, the orthodromic transposition, without changing its direction, prevents production of a soft-tissue protrusion overlying the zygomatic arch if the muscle is reflected over the arch of the zygoma, altering the dynamics of contraction, which may result in ischemia and decreased contractile strength. 15 However, modified techniques of temporalis muscle transposition have been developed, including the use of slips of temporalis fascia passed to reach the contralateral paramedian plane. 16

Good aesthetic and functional results were achieved. The patient recovered her facial symmetry and is very pleased with the final result. LTM is a good option in cases of long-standing facial paralysis, or in cases of subacute facial paralysis, if the patient desires a single-stage procedure with almost immediate dynamic function. Moreover, this technique assumes extreme importance in facial reanimation of patients who are of advanced age or those who have several comorbidities. The objective analysis in our patient suggests that it may also be considered as first reconstructive option in younger and healthy patients.
